# Immunohistochemical testing for *Helicobacter Pylori* existence in neoplasms of the colon

**DOI:** 10.1186/1471-230X-8-35

**Published:** 2008-08-14

**Authors:** Aliye Soylu, Selvinaz Ozkara, Halil Alıs, Kemal Dolay, Mustafa Kalaycı, Nurgul Yasar, A Baki Kumbasar

**Affiliations:** 1 Department of Gastroenterology, Bakirkoy Dr Sadi Konuk Training and Research Hospital, Istanbul, Turkey; 2 Department of Pathology, Haydarpasa Numune Training and Research Hospital, Istanbul, Turkey; 3 Department of General Surgery, Bakirkoy Dr Sadi Konuk Training and Research Hospital, Istanbul, Turkey; 4 Department of Internal Medicine, Bakirkoy Dr Sadi Konuk Training and Research Hospital, Istanbul, Turkey

## Abstract

**Background:**

*Helicobacter pylori* is a common pathogen, and its prevalence varies with socioeconomic conditions (10–80%). It has recently been recognized as a class I carcinogen in relation to gastric cancer. The aim of this study was to investigate the presence of *Helicobacter pylori* in neoplasms of the colon by immunohistochemical methods.

**Methods:**

The polypectomy materials of 51 patients (19 male and 32 female) who had undergone colonoscopic polypectomy were retrieved for retrospective examination. The endoscopic size and colonic localization of the polyps were recorded. Hematoxylin and eosin stains were evaluated according to histological type and grade of dysplasia. Biopsy stains were immunohistochemically treated with *Helicobacter pylori* antibodies by the streptavidine-biotin immunoperoxidase technique. *Helicobacter pylori* staining in the gastric mucosa was used as the control for the immunohistochemical method. Specimens were classified according to the presence of *Helicobacter pylori* under an optical microscope, and *Helicobacter pylori* positive specimens were stratified according to the respective staining pattern.

**Results:**

Mean age was 61.88 ± 10.62 (40–82) years. Polyp sizes were 1.45 ± 0.92 (1–4) cm; and 25.5% of polyps were localized in the right colon, 68.6% in the left colon and 5.9% in the transverse colon. Presence of *Helicobacter pylori* was not correlated with localization (p > 0.05) or size of the polyps (p > 0.05).

Eleven (21.6%) of all specimens included in the study were *Helicobacter pylori* positive by immunohistochemical methods. Of the *Helicobacter pylori* positive specimens, the staining pattern was diffuse: Equivocal in 90.9%, nonspecific with a finely granular type concentrated on the luminal surface in 90.9%, dot-like granular in 54.5%, and spiral in 9.1%. Of the tubular polyps, 17.9% were *H. pylori* positive, and the staining pattern was equivocal in 100%, luminal in 85.7%, and dot-like granular in 57.1%. Of the villous polyps, 60% were *H. pylori* positive, and the staining pattern was inconclusive in 66.7%, luminal in 100%, dot-like granular in 33.3%, and spiral in 33.3%. Of the cancerous cases, 25% were *H. pylori* positive and showed an equivocal, luminal, and dot-like granular staining pattern. No significant correlation was determined between histologic types and prevalence of *H. pylori* (p > 0.05).

**Conclusion:**

The presence of *H. pylori* in colon polyps did not yield any correlation with polyp size, colonic localization or histopathologic type. The higher rate of *H. pylori* positivity in villous polyps does not present a causal relationship. We were able to determine *H. pylori* existence in colon polyps by immunohistochemical methods, albeit with no statistical significance.

## Background

*Helicobacter pylori* (*H. pylori*) is a class I carcinogen giving rise to gastric adenocarcinoma [1,2]. In humans, apart from the gastric mucosa, it has been isolated from cholestatic liver parenchyma [3]. Experimental studies have demonstrated a relationship between certain Helicobacter species with inflammatory bowel disease and colonic adenocarcinoma development [4-6]. *H. pylori* infections have been considered as a risk factor for development of colorectal neoplasms (CRNs) such as colon polyps and colon cancer (CC) due to the high prevalence of serologically positive *H. pylori* infection among CRN patients in some uncontrolled studies [4,7,8]. Nonetheless, data pertaining to the association between CRN and *H. pylori* is limited and insufficient. Even though there exist human studies that support [7,9] or reject [10,11] a relationship between CRN and *H. pylori*, a direct colonization of the colon by *H. pylori* that would suggest a causal role for CRN has not been shown [4,7,8]. In this study, the presence of *H. pylori* in CRNs, and a potential relationship with histopathologic types of the neoplasms by immunohistochemical (IHC) methods specific to *H. pylori* was investigated.

## Methods

**Figure 1:**
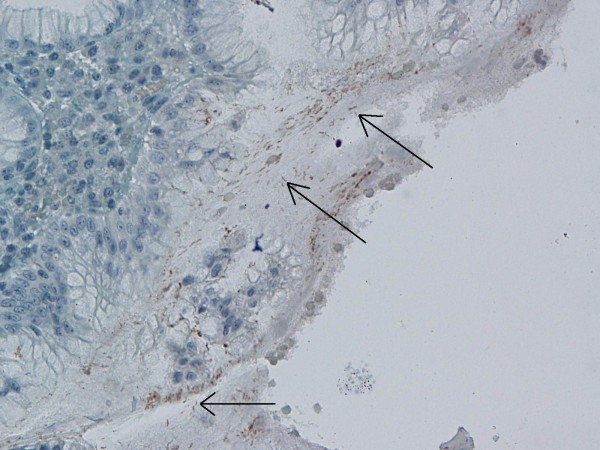
Immunohistochemical *H. pylori* positivity in gastric control biopsies (×40).

**Figure 2:**
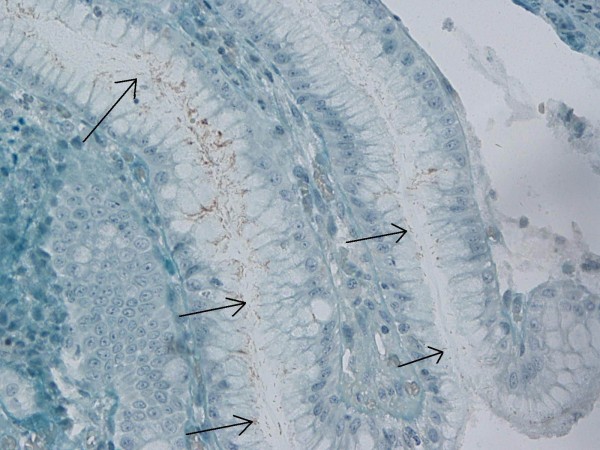
Immunohistochemical *H. pylori* positivity in gastric control biopsies (×40).

Biopsy specimens of 51 patients (19 female and 32 male) who had been administered polypectomy following detection of polyps by colonoscopy were included in the analysis. Pseudopolyps in inflammatory bowel diseases, inflammatory polyps, juvenile polyposis syndrome, familial adenomatous polyposis and colon neoplasms that did not appear polypoid by endoscopy or colon carcinomas were excluded. Polyps in all colonic localizations were included. Polyps localized in the descending colon, sigmoid colon and rectum were classified as 'left colon', those in the transverse colon as 'transverse colon' and those in the cecum and ascending colon as 'right colon' localization. The mean age of the patients was 61.88 ± 10.62 years (range: 40–82 years). Endoscopic polyp size and localization were categorized. Haematoxylin and eosin (H&E) stains obtained from paraffin blocks were retrospectively evaluated by two pathologists and classified according to histopathologic type and degree of dysplasia. In the IHC analysis following this assessment of the H&E stains, specimens were treated with *H. pylori* antibodies (ready to use, polyclonal, Biogen, Union City, CA, USA) by the streptavidine-biotin immunoperoxidase technique. Specimens were incubated with primary antibody at a dilution of 200 μg/ml at room temperature for twenty minutes [12]. The control testing for the IHC method consisted of *H. pylori* staining in the gastric mucosa (Figure 1, 2). *H. pylori* positive specimens were separated according to the IHC staining pattern of *H. pylori* under optical microscopy. An institutional ethics committee approval was obtained prior to initiation of the study.

### Statistical Analysis

To perform the statistical analyses, descriptive statistical methods, the Kruskal-Wallis test, the Mann-Whitney U test, and the chi-square test were used. Results were evaluated at a 95% confidence interval; and a p < 0.05 value was recognized as the statistical significance level.

## Results

During endoscopic investigation, polyp sizes were 1.45 ± 0.92 cm (range: 1–4 cm), 25.5% of the polyps were localized in the right colon, 68.6% in the left colon and 5.9% in the transverse colon. *H. pylori* positivity/negativity in the polyps was not correlated to localization in the colon (p > 0.05) or polyp size (p > 0.05). The distribution of histopathologic types was 76.5% tubular, 9.8% villous, 5.9% tubulovillous, and 7.8% adenocarcinoma. Of the 39 tubular cases, 38 (97.4%) had low grade dysplasia (LGD) and 1 (2.6%) high grade dysplasia (HGD); of the 5 villous polyps, 1 (20%) had LGD and 4 (80%) HGD; and finally, all 3 (100%) of the tubulovillous polyps had LGD. Adenocarcinoma was established in four polyps.

**Figure 3:**
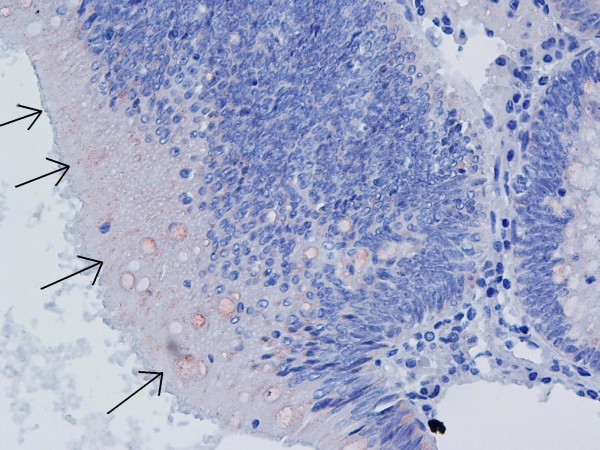
Nonspecific finely granular staining concentrated on the luminal surface by IHC staining (×40).

**Figure 4:**
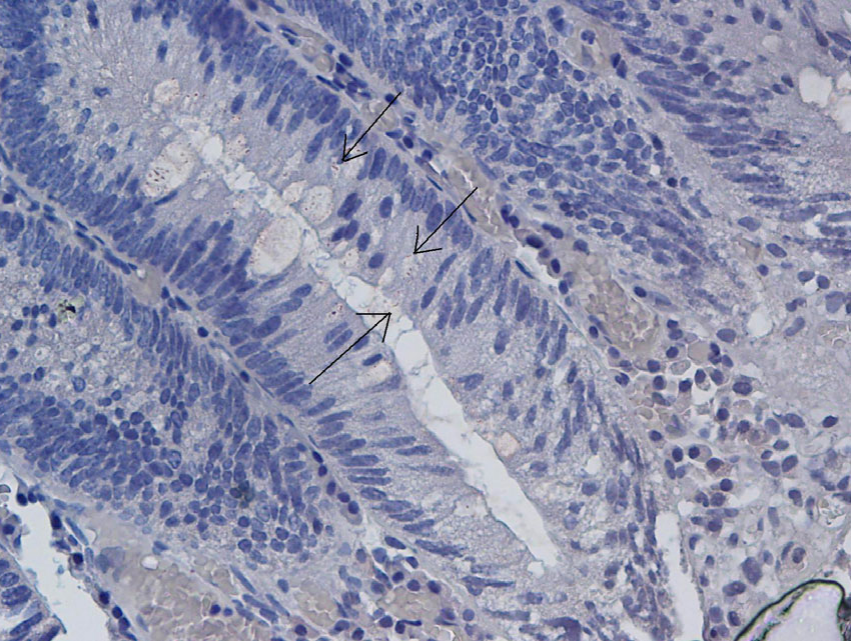
Spiral-form dark yellow/light brown staining in lumen of adenomatous polyp (×40).

**Figure 5:**
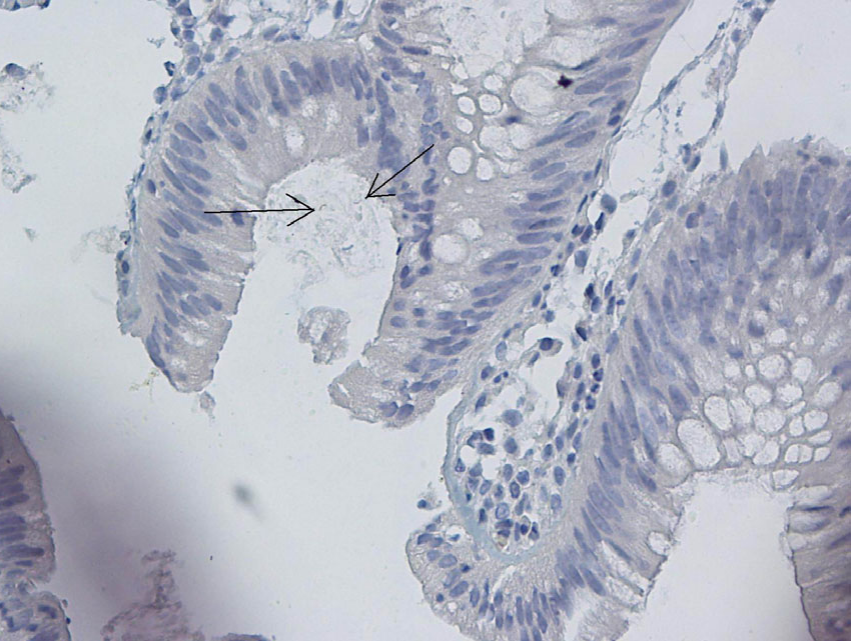
Spiral-form dark yellow/light brown staining in lumen of adenomatous polyp (×40).

11 (21.6%) of the polyps were determined to be *H. pylori* positive by IHC evaluation. Of these 11 specimens, 10 (90.9%) had an equivocal diffuse mucosal staining pattern, 10 (90.9%) had nonspecific finely granular staining concentrated on the luminal surface (Figure 3), and 6 (54.5%) also had a dot-like granular staining pattern. The weak dark yellow/light brown staining in a spiral form in 1 (9.1%) specimen was evaluated as positive (Figure 4, 5). Staining patterns of *H. pylori* positive specimens according to histopathological polyp types are summarized in Table 1.

**Table 1:** Positivity for *H. pylori* and staining patterns according to histopathologic type

	Tubular (n = 7)	Villous (n = 3)	Cancer (n = 1)
	n (%)	n (%)	n (%)
*H. pylori* presence (p = 0.137)			
Positive	17.9%	60.0%	25.0%
Negative	82.1%	40.0%	75.0%
Staining patterns of *H. pylori* (+) specimens			
Equivocal	7 (100%)	2 (66.7%)	1 (100%)
Luminal	6 (85.7%)	3 (100%)	1 (100%)
Dot-like Granular	4 (57.1%)	1 (33.3%)	1 (100%)
Spiral	-	1 (33.3%)	-

The prevalence of *H. pylori* was higher in villous type polyps (60%) compared to other histologic types, but this difference was not statistically significant. We were not able to establish a significant correlation between the polyps' histologic types and the prevalence of *H. pylori* (p > 0.05).

## Discussion

Epidemiological studies have confirmed a causal relationship between *H. pylori* and gastric cancer [13,14] and colonic phenotype of *H. pylori* related intestinal metaplasia (IM) has been associated with gastric cancer [1,15]. Thus, association of *H. pylori* in various gastrointestinal system organ cancers has been investigated and Helicobacter DNAs were positive in 52.6% of the hepatobiliary cancer cases. This positivity suggests that Helicobacter species may play a role in the pathogenesis of hepatobiliary cancer through an acceleration of biliary cell kinetics [16]. Helicobacter species, which may colonize the biliary tract, have been implicated as a possible cause of hepatobiliary diseases ranging from chronic cholecystitis and primary sclerosing cholangitis to gall-bladder carcinoma and primary hepatic carcinomas [17]. Therefore the hypothesis that *H. pylori* would also be associated with intestinal polypoid structures needs to be investigated. Furthermore, there exist several studies demonstrating the co-existence of CRNs and *H. pylori* seropositivity supports this suggestion. The potential mechanism of the significant association between colon polyps and serologic *H. pylori* positivity has been attributed to the remote trophic effect of the elevated gastrin level on the colonic mucosa [9]. This study has been designed as to investigate an association between *H. pylori* and extragastric intestinal neoplasms and colonization in cases of direct colonic dysplasia has been investigated by specific IHC methods.

Shmuely et al. reported that *H. pylori* CagA+ seropositivity is enhanced in gastric and colon cancers [18], while Fireman et al. demonstrated a correlation between *H. pylori* seropositivity and CA19-9 elevation in patients with CC [19]. In the evaluation by Mizuno et al. of the colon pathologies of 332 patients with high-resolution colonoscopy, the increase in the incidence of adenomatous polyps in *H. pylori* IgG seropositive patients and diminution of normal colonoscopy findings was found more significant than in seronegative patients [20]. While the underlying mechanism is not clear, the prevalence of gastric *H. pylori* infection was found to be increased, especially in colonic adenomas (71.4% in polyps, 55% in cancers) [21]. In case reports of Cap polyposis that support the correlation between CRN and *H. pylori*, despite the inability to demonstrate *H. pylori* in polyps by IHC methods, eradication of the infection led to improvements in symptoms and polyps [22,23]. Although direct and indirect relationship between *H. pylori* and CRNs have been widely recognized, only the relationship between the presence of serologically positive *H. pylori* and CRNs have been demonstrated [4,8]. It has also been reported that the remote and local consequences of *H. pylori* infection might also have a synergistic effect on the emergence or development of these neoplasms under certain conditions [4,8]. A study of 374 patients with GI cancers evaluated serologic *H. pylori* positivity according to localization, serologic *H. pylori* positivity was found to be unrelated to CC localization [24]. In an investigation for *H. pylori* specific 16S rDNA with PCR in CC biopsies, Grahn et al. revealed *H. pylori* DNA in 27% of cancer tissue specimens. They could not ascertain any correlation between *H. pylori* positivity and the stage or colonic localization of the cancer [25]. We also found no association with the size or colonic localization of polyps determined to be *H. pylori* positive by IHC methods.

In a study by Bulajic et al. on 83 subjects with CC that investigated *H. pylori* DNA by PCR in biopsy specimens from CC and normal mucosal tissues, *H. pylori* IgG antibody was positive in 36 patients while *H. pylori* was determined by PCR in the tissues of 1 patient with CC and 5 specimens of normal mucosa. However, no correlation between *H. pylori* positivity and CC could be demonstrated [10]. There exist serologic and colon tissue PCR studies that support [11,20,26] or reject [5,10,27] a correlation between *H. pylori* and CRNs.

The Giemsa staining method is specific for the determination of gastric spiral forms of the organism. However, IHC methods have been found to better identify non-spiral forms of the bacterium [7,28-32]. It has been suggested that a dot-like staining pattern with the IHC method represents the coccoid form of *H. pylori*, and this method of staining makes it impossible to differentiate mucus or debris on the lumen from antigenic structures with *H. pylori*-like reactions or stain precipitates. Although IHC staining is more specific than the Giemsa method routinely used for the evaluation of gastric *H. pylori*, false positives may be difficult to conclusively differentiate [7,28,29]. In our study, *H. pylori* showed various staining patterns with IHC method. Therefore we regarded a dot-like granular staining pattern as a positive finding together with the coexistence of other luminal or equivocal staining patterns.

Similar to our study, in a trial investigating presence of *H. pylori* in colon polyps by IHC methods, *H. pylori* was determined in tubular and tubulovillous adenomas, but not in villous polyps. This result was deemed insignificant and interpreted to be potentially related to the micro-environment [7]. Similarly, as with the low prevalence of *H. pylori* in gastric IM sites or some gastric fundic polyps, the micro-lining in villous adenomas may also be inopportune for *H. pylori*[7,33]. Contrary to this finding, we found a higher prevalence of *H. pylori* positivity in villous polyps compared with other histologic types. A similar situation is present within studies investigating presence of *H. pylori* in gastric IM [34,35]. Whilst some studies support the assumption that *H. pylori* is absent in gastric IM; in studies using IHC method presence of *H. pylori* in gastric IM have been demonstrated [28,33,36]. Furthermore, the absence of *H. pylori* in villous adenomas as reported by several authors does not necessarily indicate the lack of an association, as this might be supportive of the hypothesis about *H. pylori* migration after development of the lesion [7,28,37]. Despite reports of positivity for *H. pylori* in tubular and tubulovillous adenomas [7] and the lack of a significant association with cellular types of polyps in our study, reports that have directly demonstrated the presence of *H. pylori* in villous adenomas (60%) by IHC methods exist in medical literature. The small number of materials in our study may account for the lack of statistical significance. However, the lack of a correlation between cellular types of polyps and *H. pylori* presence indicates that the micro-environment may indeed have a role, and *H. pylori* strains may vary.

## Conclusion

This study has demonstrated the presence of *H. pylori* in colon polyps by IHC methods, albeit with no statistical significance. Our findings do not suffice for the assertion of a definitive association between *H. pylori* positivity and CRNs. However, even in the absence of a causal relationship, our results are suggestive of a correlation between colon polyps and *H. pylori*. Further cellular studies that are supported by molecular biological techniques are needed to clarify the presence or absence of such an association.

## Abbreviations

CC: colon cancer; CRN: colorectal neoplasm; GI: gastrointestinal; H&E: haematoxylin and eosin; HGD: high grade dysplasia; *H. pylori*: *Helicobacter pylori*; IHC: immunohistochemical; IM: intestinal metaplasia; LGD: low grade dysplasia.

## Competing interests

The authors declare that they have no competing interests.

## Authors' contributions

AS planned and coordinated the study, and prepared manuscript; SO prepared and stained specimens, and evaluated specimens; HA, KD and MK were involved in the compilation of colonoscopic polypectomy cases; NY was involved in data entry; ABK was involved in literature search. All authors read and approved the final manuscript.

## Pre-publication history

The pre-publication history for this paper can be accessed here:


